# Pseudogenes regulate parental gene expression *via* ceRNA network

**DOI:** 10.1111/jcmm.12952

**Published:** 2016-08-25

**Authors:** Yang An, Kendra L. Furber, Shaoping Ji

**Affiliations:** ^1^ Department of Biochemistry and Molecular BiologyMedical SchoolHenan UniversityHenan ProvinceChina; ^2^ College of Pharmacy and NutritionUniversity of SaskatchewanSaskatchewanSKCanada

**Keywords:** gene expression, pseudogene, microRNA, ceRNA

## Abstract

The concept of competitive endogenous RNA (ceRNA) was first proposed by Salmena and colleagues. Evidence suggests that pseudogene RNAs can act as a ‘sponge’ through competitive binding of common miRNA, releasing or attenuating repression through sequestering miRNAs away from parental mRNA. In theory, ceRNAs refer to all transcripts such as mRNA, tRNA, rRNA, long non‐coding RNA, pseudogene RNA and circular RNA, because all of them may become the targets of miRNA depending on spatiotemporal situation. As binding of miRNA to the target RNA is not 100% complementary, it is possible that one miRNA can bind to multiple target RNAs and vice versa. All RNAs crosstalk through competitively binding to miRNA *via* miRNA response elements (MREs) contained within the RNA sequences, thus forming a complex regulatory network. The ratio of a subset of miRNAs to the corresponding number of MREs determines repression strength on a given mRNA translation or stability. An increase in pseudogene RNA level can sequester miRNA and release repression on the parental gene, leading to an increase in parental gene expression. A massive number of transcripts constitute a complicated network that regulates each other through this proposed mechanism, though some regulatory significance may be mild or even undetectable. It is possible that the regulation of gene and pseudogene expression occurring in this manor involves all RNAs bearing common MREs. In this review, we will primarily discuss how pseudogene transcripts regulate expression of parental genes *via* ceRNA network and biological significance of regulation.

## Introduction

miRNAs are short 19‐23 nt of RNA transcribed from the endogenous genome and are found distributed throughout the cells 1. After transcription, pri‐miRNA or pre‐miRNA needs to be processed into mature microRNA. Differing from the other regulation (such as transcriptional activation or repression), miRNAs finely control gene expression primarily by repressing translation and/or promoting degradation target RNAs depending on complementary seed length in miRNAs. RNA degradation occurs when miRNA has a high complementary grade to the target RNAs. Considering homolog of pseudogenes with parental genes, it is possible that pseudogene transcripts can serve as ceRANs to regulate parental RNAs. For a given cell, the total transcripts containing similar miRNA response elements (MREs) can theoretically regulate each other *via* competitive binding to common miRNA, and as such serve as miRNA sponge 2, 3. Competitive capacity generated by absorbing common miRNA results in decreased miRNA availability or reduced free miRNA levels. This releases miRNA repression on parental mRNA, dynamically regulating mRNA translation speed or mRNA stability.

Competitive endogenous RNA (ceRNA) are comprised of protein‐coding RNA, tRNA, rRNA, long non‐coding RNA (lncRNA), pseudogene RNA and circular RNA 4. Most ceRNAs have potential MREs, share common miRNAs and compete for binding common RNAs according to ceRNA theory 3. As ceRNA, lncRNA HULC(highly up‐regulated in liver cancer) serves as a miRNA sponge, plays an important role in regulation of cancer‐related genes. It absorbs miR‐372 and releases repression of miR‐372 on PRKACB translation. PRKACB can induce phosphorylation of CREB which control HULC transcription 5. It is likely necessary to keep stable/balance of the feed‐back loop. A disruption such as HULC aberrant may contribute to tumourigenesis.

Pseudogenes are relicts of parental genes, through replication and mutation during evolutionary process they have lost the function of encoding for full‐length functional proteins 6, 7. Increasing evidence indicates that pseudogenes are a critical part of the complex, multi‐tier regulatory network governing gene expression 8. A systematic analysis of pseudogene transcripts shows that pseudogenes are genome‐widely transcribed from 293 samples including 13 types of cancer and normal tissue. Some of the pseudogenes could be categorized into different groups, representing cancer specific, lineage specific or ubiquitously expressed pseudogenes 9. This arises a possibility that the pseudogene transcripts can be developed into a bio‐marker for cancer diagnosis in future. In addition to competing for binding of miRNAs, some truncated peptides encoded by pseudogenes may still play functional roles 10, 11, 12. Pseudogenes have defined roles in regulating gene expression, and therefore should be considered as ‘real’ genes. Recent studies show numerous transcripts, including pseudogene RNAs, can up‐regulate target gene expression through competing for binding of common miRNAs which attenuates miRNA repression of target genes 13, 14, 15. In this review, we mainly discuss potential relationship between pseudogenes and parental based on proposed ceRNA network. It is possible that pseudogenes play other biological function along further investigation.

Djebali *et al*. collected data from 15 cell lines and reported that 74.7% of human genome was primarily transcribed 16. It is estimated that ~95% of human genome is transcribed under a variety of spatiotemporal conditions, but protein‐coding genes only account for ~2% of the genome 17. Furthermore, bi‐direction transcription and transcription pausing of promoter yield various lengths of RNA that can be spliced into coding RNA and lncRNA 18. This indicates that non‐protein‐coding RNAs may account for major portion of total RNA and constitute a complex network of competition *via* miRNAs. According to GENCODE Release (version 24) (http://www.gencodegenes.org/stats/current.html), there are 14,505 pseudogenes in human genome, but it is still unclear whether these pseudogenes spatiotemporally transcribed. Han *et al*. identified 9925 pseudogene transcriptions from 7 type cancer samples of 2808 patients 19. It seems to be difficult to distinguish pseudogene transcription from other long non‐coding RNAs, so exact number of pseudogene transcriptions has not been determined. Evolution of lncRNA and pseudogenes have been well reviewed 20. Both lncRNA and pseudogene transcripts are able to regulate expression of protein‐coding genes at diverse levels. Here, we focus on the progress that has been made on understanding how pseudogenes regulate the expression of parental genes *via* ceRNA network involved in tumourigenesis.

## The relationship between pseudogenes and parental genes

As protein‐coding genes account for only ~2% of human genome, the other ~95% is often referred a ‘junk DNA’ that is evolutionarily remnants. However, recent studies indicate that most of this junk DNA is transcribed under different spatiotemporal conditions. As a result, apart from tRNA and rRNA, most transcripts are non‐coding which include lncRNA, miRNA, pseudogene RNA and circular RNA.

Pseudogenes are remnants of their parental genes that lost encoding function as a result of gradual mutation 21, such as mutations in regulatory elements and encoding regions. It is believed that amplification of genes also occurred during evolution, in which the copy number of a given gene expanded 22. Some genes may not have been used for coding protein for a significant period of time leading to numerous mutations and degeneration into pseudogenes. At regulation of transcription level, lncRNA mainly recruit epigenetic modifiers to DNA, leading transcription silence or activation in genome 23, 24, but pseudogenes most likely regulate parental gene expression *via* binding of shared miRNAs. Regulation of pseudogene expression at epigenetic level, such as DNA methylation, appears to be independently established apart from parental genes. However, the level of DNA methylation significantly depends on the status of local DNA micro‐environment 25. Therefore, it is possible that pseudogenes are not passively remained in revolution. Conversely, they may be positively selected to remain and play some roles in gene expression and regulation.

Pseudogene transcripts can generate non‐coding RNA and anti‐sense RNA that act as RNA sponges for miRNA 26. Recent investigation showed that PTEN pseudogene PTENP1 up‐regulated expression level of PTEN by competing for binding to miRNAs shared with PTEN 27. As a result, it released PTEN repression in a DICER‐dependent manner 13. Interestingly, two anti‐sense lncRNA (asRNAα and asRNAβ) from a locus of pseudogene PTENP1 also modulate PTEN expression. The asRNAα binds to PTEN promoter region recruiting DNMT3a and EZH2 to epigenetically down‐regulate PTEN transcription. However, asRNAβ binds to PTENP1 transcript and stabilizes PTENP1, which serves as ceRNA of PTEN. Consequently, asRNAβ up‐regulates *in trans* PTEN expression *via* miRNA and PTENP1 28. Anti‐sense transcription may also occur for other pseudogenes. Pseudogene FLT1P1 of VEGF receptor‐1 (VEGFR1) regulates parental gene VEGFR1, and intriguingly, expression of FLT1P1 anti‐sense can inhibit both expression of VEGFR1 and VEGF‐A likely *via* ceRNA. Knock‐down of FLT1P1 expression represses tumour cell proliferation. Mechanistically, FLT1P1 may share common miRNA through ceRNA network 29. Another anti‐sense pseudogene transcript of Oct4 may epigenetically down‐regulate expression of Oct4, Oct4 and Oct5 pseudogenes 30. It suggests pseudogene transcripts of both sense and anti‐sense may involve in epigenetic regulation of target genes.

Similarly, human cytochrome P450 gene CYP2A6 and its pseudogene CYP2A7 compete for binding of miR‐126* and up‐regulate expression of each other 31. These findings support that pseudogene transcripts can up‐regulate expression of the protein‐coding genes most likely by competing for shared miRNAs. As a category of lncRNA, pseudogene transcripts most likely play similar functional roles on expression of their parental genes *via* proposed ceRNA network. It is well‐documented that 3′UTR of mRNA is main target of microRNAs that regulate gene expression by repressing translation 32. Evidence shows that overexpression 3′UTR of pseudogene CYP4Z2P increased CYP4Z1 expression 33, probably by acting through the ceRNA network. Thus, the primary function of pseudogene transcripts is the regulation of parental gene expression through sequestering common miRNAs and releasing expression inhibition.

In addition, pseudogene transcripts are able to down‐regulate expression of parental genes *via* other mechanisms. Oct4 pseudogene Oct4P4 forms a complex with SUV39H1 HMTase and HP1α and epigenetically down‐regulates expression of parental gene Oct4, leading repression of mouse stem cell self‐renewal 34. In this situation, the pseudogene acts similarly to lncRNAs that participate in forming a complex with other RNAs and genomic modifiers to epigenetically modulate DNA transcriptional activity.

Furthermore, transcripts of some pseudogenes encode shortened peptides which may also be involved in regulating parental gene expression. Although CLRX.1/NOD24 pseudogene NLRP2P lacks full‐length coding region, it encodes a 45‐amino‐acid protein which is highly homologous to Pyrin‐only protein 2 (POP2/PYDC2), a regulator of NF‐kb. As a result, NLRP2P peptide inhibits transcriptional activation of NF‐kb and down regulates NF‐kb expression 10. However, it is unclear whether this regulatory role of the truncated peptide from NLRP2P pseudogene is representative of other pseudogenes. Many pseudogenes can encode a shortened peptide due to earlier stop codons, but further investigation is needed to understand their biological functions.

## Pseudogenes as competitive endogenous RNA

Increasing evidence supports the hypothesis of competitive endogenous RNA, in which RNAs that share common MREs can absorb or sequester miRNAs, attenuating and/or release suppression of miRNAs on protein‐coding RNAs. Pseudogene transcripts (as ceRNA) preserve parental gene mRNA have been further demonstrated from conserved pseudogenes in mice 35. It is well‐documented that miRNAs finely tune mRNA and protein expression levels primarily by repressing translation through binding to MRE in 3′UTR of mRNAs, and only complementary small RNA can lead target RNA degraded. Computational analyses suggest that more than 60% of human protein‐coding mRNAs contain predicted MREs 36, 37. Further quantitative experimentation revealed the regulation through the ceRNA network *via* miRNA availability mainly involved regulation of gene expression at the transcription factor level 36. Tight control of transcription factor expression may be an economical and effective model, as it would serve to regulate expression of numerous downstream targets. Quantitative analyses based on mathematical modelling has provided insight into the relationship between miRNA and ceRNA or among various ceRNAs 38; however, even with state‐of‐the‐art bioinformatics it remains difficult to predict the exact nature of miRNA and target RNA interactions 39. According to a recent study 2, ceRNA regulatory efficiency is based on the ratio of miRNA: target pool RNAs. In other words, the efficiency of miRNA repression of a target gene expression depends on the abundance of both miRNAs and target RNAs. A high abundance of target RNAs will spread the binding of miRNA across a larger pool and reduce the efficiency of miRNAs while a low abundance of target RNAs will increase the efficiency of miRNAs 40. Different algorithms have been employed to predict interactions of miRNA among common target ceRNA. For example, starBase v2.0 was used to identified 9000 potential interactions between miRNA and circular RNA, 16,000 interactions between miRNA and pseudogene RNA and 285,000 interactions between proteins and RNAs 41.

**Figure 1:**
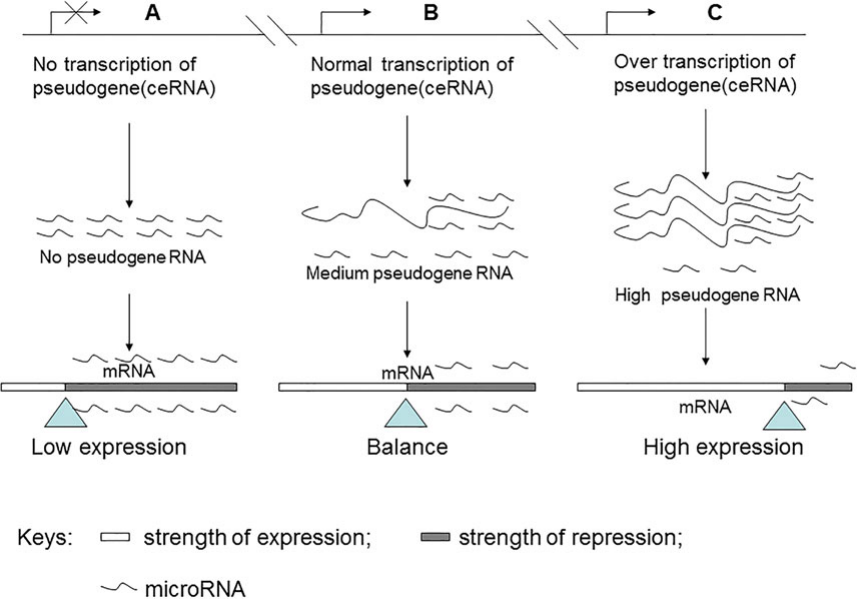
A balance between protein‐coding genes and pseudogenes (ceRNA). (**A**) In the context of little or no pseudogene transcription, expression of protein‐coding genes is significantly repressed by miRNAs that would normally be shared by all transcripts (ceRNA) but are now mainly absorbed by protein‐coding genes; (**B**) All transcripts constitute a balanced network in which protein‐coding genes are appropriately expressed; (**C**) When pseudogenes(ceRNA) are spatially and temporally overexpressed, pseudogene transcripts (ceRNA) absorb most miRNAs and released repression of protein‐coding genes. As a result, expression of the parental genes is significantly increased.

Most miRNAs have multiple targets which are conserved throughout evolution. In a ceRNA network, a change in the expression level of any ceRNA transcript level may disturb balance within the ceRNA network. Shao *et al*. employed bioinformatic methods and found that disruption of ceRNA network involved in development or progression of lung adenocarcinoma 42, indicating significance of ceRNA network balance. Potential none‐coding targets may diminish the binding of miRNAs with ‘authentic’ mRNA targets. As depicted in Figure 1, pseudogene RNAs complete for binding of miRNAs and release the repression on mRNA transcription.

Pseudogene PTENP1 of tumour‐suppressor PTEN, regulated expression level of PTEN by competing for binding of common miRNA and releasing PTEN repression in a DICER‐dependent manner 13. Among ceRNAs, circular RNAs typically play a role as miRNA sponges. Circular RNA ciRS‐7 contains approximately 70 conserved MREs, depleting the miRNA pool through sequestering miR‐7 and preventing it from binding to target mRNA 43. Similarly, circular RNA CDR1 contains 63 conserved MREs for miR‐7, acting as a miRNA sponge for miR‐7 during neuronal development 44. Ebert *et al*. developed an artificial miRNA sponge containing multiple, tandem MREs, expressed within cells. This miRNA sponge is functionally similar to some circular RNA and releases miRNA repression on mRNAs 45. Apart from serving as ceRNAs, circular RNAs may play other important roles in biological functions 4.

Extensive cross‐talk among ceRNA *via* binding of common, semi‐complimentary miRNA makes it difficult to identify the ‘regulatory efficiency’ of subsets of miRNA to target RNA. However, the complicated nature of the ceRNA network can be simplified as an autonomous module in which there is an inverse correlation in expression level between all miRNAs and all transcripts, and in contrast, there is positive correlation in expression level among all transcripts but not all miRNAs 46.

Controversially, a systematic analysis on the ceRNA hypothesis suggested that regulation through the ceRNA network occurred within a typical cellular environment *in vivo*. However, it underscored that the release target repression by competitive binding of ceRNA to miRNA needs involvement of a minimum number of MREs. This has been termed threshold‐like effectiveness, and thus low levels of MREs/ceRNA do not have competitive efficacy on each other 47. For example, miR‐122 is an abundant non‐coding small RNA in hepatocytes and its target RNAs are thought to compete for binding in accordance with the ceRNA hypothesis. In an evaluation assay, the forced expression of miR‐122 binding site needed to release repression of endogenous target genes was so high that it would never be reached under normal physiological conditions 47. These experiments revealed that competitive binding of ceRNA to common miRNA under normal expression levels is unlikely, although it may occur in some extreme contexts such as under disease condition.

Other observations indicate that only miRNAs that have high expression levels can bind to target RNA while low abundant miRNAs can not compete for binding to target RNA. Thus, ceRNA networks do not exist in most cells 48. Mullokandov *et al*. 49 predicted that only high abundant miRNAs can execute repression effects on target RNAs, which would account for less than 40% of entire miRNAs in the intact cells. It appears that a higher ratio of miRNA to target RNA generates stronger repression 49. In summary, further investigation of an extensive number of diverse cell types is necessary to determine whether there is sufficient evidence for pseudogenes regulating expression of their parental *via* a ceRNA manner.

## Dysregulation of pseudogene expression in tumourigenesis

### Pseudogene expression inhibits cancer progression

Binding of miRNA to target RNA usually is not 100% complementary, but a seed region on miRNA is necessary. In ceRNA competitive regulatory network, imperfect complementary sequences can provide even higher competitive effectiveness by more efficiently absorbing and sequestering miRNA. In fact, imperfect binding may result in miRNAs binding to MREs for a longer period of time than perfect binding through which the miRNAs are released after target degradation 50. This would have a greater propensity to reduce the repression of miRNA on target RNA, such as mRNAs.

Most ceRNAs (including pseudogenes) contain MREs that can sequester miRNAs in an imperfect match fashion. Recent studies indicated that many pseudogenes play important roles in regulating parental gene expression, especially in the expression of oncogenes and anti‐oncogenes in various cancers. It is well‐documented the pseudogene PTENP1 up‐regulated expression of PTEN in a DICER‐dependent manner 13, 51. Furthermore, promoter methylation leading to down‐regulation of PTENP1 expression facilitates proliferation and invasion of clear‐cell renal cell carcinoma, but up‐regulation of PTENP1 expression inhibits cancer cell survival 15. It seems likely that PTENP1 transcript sequesters miRNA21 (shared by PTEN) and releases repression of PTEN expression 52. Another member of the ceRNA network, lncRNA‐BGL3 represses transformation of the mouse primary bone marrow cells induced by Bcr‐Abl, which is a fusion gene involved in chronic myeloid leukaemia. Further experiments showed that lncRNA‐BGL3 and PTEN share common miRNAs, suggesting the lncRNA‐BGL3 may promote PTEN expression through binding and sequestering miRNAs that repress PTEN mRNA translation 53. The importance of the MRE‐containing 3′UTR is highlighted in the process of absorbing and sequestering miRNAs. For example, both tumour suppressor candidate 2 (TUSC2) 3′UTR and its pseudogene can inhibit cancer cell proliferation and survival *via* competitive binding of miRNAs which results in enhanced TUSC2 translation 54. Suppression of cancer *via* ceRNA occurs between different genes as well. FOXO1 3′UTR shares miR‐9 with E‐cadherin. As a result, overexpression of FOXO1 3′UTR enhanced E‐cadherin expression, and in turn inhibited metastasis activity in breast cancer cells 55. Similarly, forced expression of the pseudogene Foxo3P, Foxo3 circular RNA and Foxo3 mRNA all could suppress tumour growth and cancer cell proliferation and survival 56.

Furthermore, attenuation of onco‐miRNAs effectiveness can reduce risk of cancer development. Both INTS6 and pseudogene INTS6P1 play roles in repressing hepatocellular cancer by competing for onco‐miR‐17‐5p binding, which facilitates hepatocellular cancer initiation and progression 57.

To evaluate the potential function of pseudogenes in tumours, the transcription level of 440 pseudogenes was measured in breast cancer biopsies. Among them, the expression level of 309 pseudogenes was significantly altered. Intriguingly, the transcription level of pseudogenes can be used to distinguish cancer samples from control samples 58. Expression level of the pseudogenes have a positive correlation with parental genes, indicating the pseudogenes may play a ceRNA role in sequestering miRNA and release repression of mRNA 58, 59. This makes pseudogenes attractive candidates as biomarkers for cancer diagnosis and prognosis, although these preliminary findings must be further validated.

### Pseudogenes facilitates cancer progression

Regulation of gene expression by ceRNA can result in both anti‐oncogene and oncogene effects in tumourigenesis. The oncogene *raf* is necessary for activity of Mitogen‐activated protein kinase (MAPKs) and is implicated in diverse cancer development by maintaining the activated status of MAPKs. In engineered mouse models overexpressing the full‐length of B‐Raf pseudogene Braf‐rs1, or its coding sequence or its 3′UTR, animals developed malignant tumours similar to human diffuse large B cell lymphoma. Subsequent analyses indicated that pseudogene expression enhanced B‐Raf expression *via* the ceRNA network by releasing miRNA repression on B‐Raf 60. Similarly, overexpression of versican 3′UTR can up‐regulate expression of versican isoforms V0 and V1, which enhance the development of hepatocellular carcinoma *in vivo* and promote tumour phenotype of HepG2 cells *in vitro* 61.

High Mobility Group A (HMGA), also known as ‘architectural transcription factors’, is often highly expressed in pituitary tumours. The pseudogenes HMGA1P6 and HMGA1P7 serve as ceRNAs which up‐regulate HMGA expression facilitating proliferation and migration in pituitary tumour cell line 62, 63. In addition, pseudogenes may be involved in regulating the aberrant expression of parental genes. OCT4 is a marker of undifferentiated cells and is aberrantly expressed in many types of cancer. OCT4‐pg4 is the pseudogene of OCT4 and is also highly expression in diverse human cancers. OCT4‐pg4 can function as a miRNA sponge competing for binding of miR‐145. This up‐regulates expression of OCT4 and induces cancer initiation 64. Furthermore, high expression of another OCT4 pseudogene POU5F1B (POU domain class 5 transcription factor 1B) enhances cancer cell proliferation and tumuorigenesis in gastric cancer 65.

## Complexity of gene expression regulation

The complexity of molecular networks governing gene expression is attracting much attention to the multiple levels of regulation that exist within a cell, including an emerging key role for pseudogene/ceRNA. For a given protein‐coding gene, the regulation of its transcription, splicing, translation and stability occurs through various different mechanisms. Regulation of gene expression by competing RNAs *via* miRNA adds a new layer of complexity 6, which will provide us new insight into understanding the relationship between gene regulatory networks and disease development. Along with more the established roles for lncRNA and circular RNA, pseudogenes appear to serve as important players in the ceRNA network.

In a stable network, expression levels of all transcripts are delicately controlled. Any increase or decrease of an individual transcript in the network may perturb this balance 66 and a new stability will need to be re‐established. A sternly controlled network is necessary to avoid severe perturbation that will lead pathological conditions such as cancer 67. Pseudogenes are likely to be simultaneously transcribed with their parental genes and play important ‘buffer’ roles in regulating parental gene expression. Except sense transcription of pseudogenes, anti‐sense transcripts of pseudogenes also play some functional roles, such as PTENP1, both asRNAα and asRNAβ and asOct4p can regulate their parental gene expression 28. Other anti‐sense transcripts of pseudogenes are supposed to be identified and determined function. To keep expression of all transcripts in a moderate and appropriate level, a regulation system should be elaborately designed and operated.

Pseudogenes usually show high homology to their parental genes in sequence, which confers the capacity to potentially bind common miRNA. In any one ceRNA, there may be more than one MRE for a given miRNA, but seed anti‐sequence of MRE may be subtly different. Therefore, affinity of miRNA to MRE will vary depending on length or number of complementary base pairs, which results in alternate strength of regulatory efficiency. Competition of ceRNA binding to a given miRNA will produce diverse effects on ceRNA themselves, including stability and translation speed 51. Finally, the subcellular localization of ceRNA and miRNA will also dictate their ability to interact and ultimately exert their effect on gene expression. To date, there is still much we do not understand about ceRNA networks which are comprised of all transcripts expressed at any given time in different cell types and/or at different cellular stages.

## Extention of the ceRNA network

**Figure 2:**
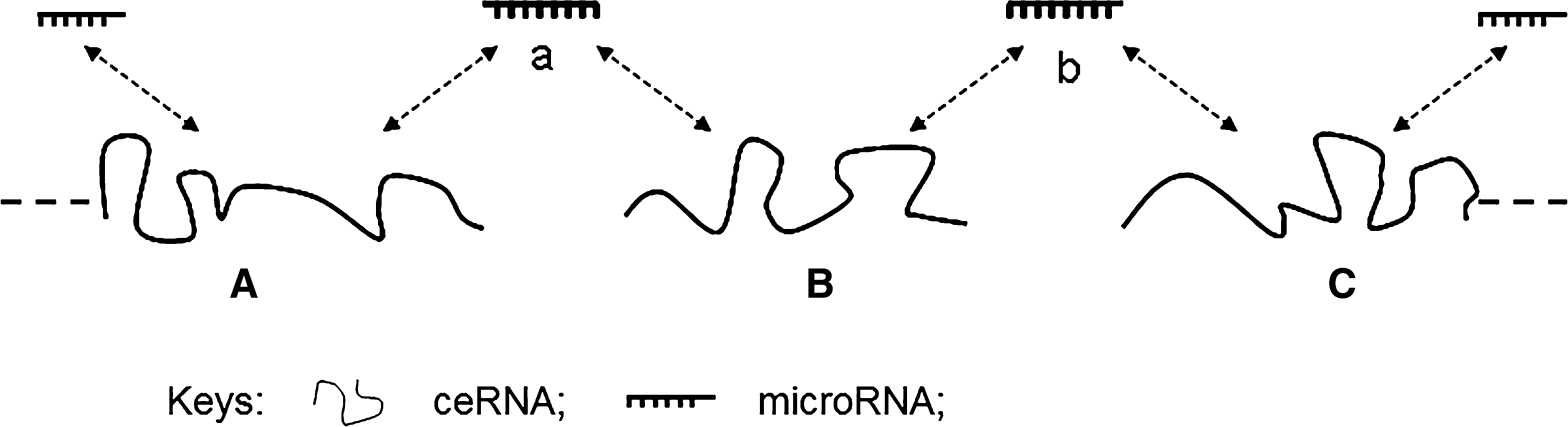
Interactions between ceRNAs *via* miRNAs. Transcripts A and B, such as parental genes and pseudogenes, share the common miRNA‐a, and would have a strong regulatory effect on each other. In contrast, transcripts A and C do not share the same miRNA, but they have indirect contact *via* transcript B, so transcript A and C would have a weak regulatory effect on each other. If A dominantly adsorbs miRNA‐a, so B will be able to adsorb more miRNA‐b. As a result, repression of miRNA‐b on C will be reduced. Thus, a regulatory interaction between A and C will occur *via* a ceRNA chain. As an extention, the farther distance between ceRNAs in the chain, the weaker the regulatory effect would be on each other. Further complexity is added by imperfect complementary binding between miRNAs and diverse MREs located on ceRNAs in the entire network.

Theoretically, as one miRNA may have more than one target RNA, all transcripts (including pseudogene transcripts) bearing MREs can connect through ‘ceRNA‐miRNA‐ceRNA‐miRNA‐’ chain and form a network (Fig. 2). ceRNAs sharing a common miRNA may interact directly through the miRNA. However, the ceRNAs that do not share a common miRNA may indirectly connect together through a chain of ‘ceRNA‐miRNA‐ceRNA‐miRNA‐’ (Fig. 2). Pseudogene transcripts can at least consolidate the ceRNA network.

**Figure 3:**
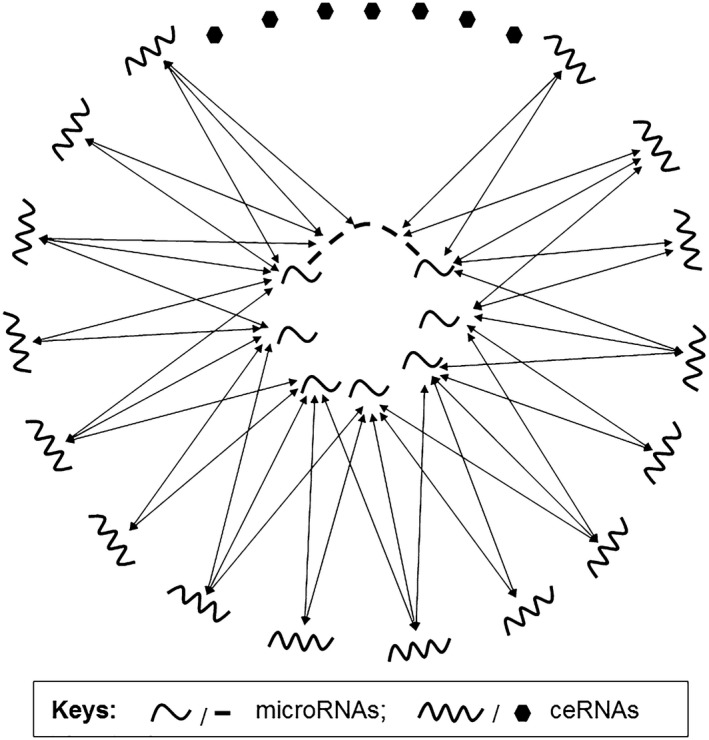
Formation of a ceRNA network. As proposed by Salmena *et al*. 3, all transcriptional RNAs may form an entire network *via* miRNAs in a given cell. Pseudogene transcripts constitute a part of this entire network and play regulatory roles in controlling parental gene expression. As each ceRNA transcript may have more than one MREs and each miRNA may have multiple target sites, a ceRNA network will form *via* interaction between miRNAs and long RNAs.

In general, the entire ceRNAs and miRNAs will form a network in which all ceRNAs and miRNAs either directly or indirectly connect with each other (Fig. 3). The ceRNAs sharing a common miRNA compete with each other for binding to miRNA and have a strong influence on their expression. Pseudogene is likely to strongly influence parental gene, because of their high similarity in sequences. In contrast, the ceRNAs that do not share the same MRE will compete weakly and yield a lower regulatory effect on each other. Since the whole network is so massive, some regulatory effectiveness among some ceRNAs will be too weak to be detected. Biological function of the emerging pseudogenes are still to be identified, merging into a pool of ceRNAs bearing MREs that are recognized and bound by common miRNAs. In the future, a better understanding of such networks or local sub‐cellular networks will provide critical insight into the important relationship between Pseudogene and parental gene expression.

We must also consider the differences in MRE affinity for a given miRNA in cells. High‐affinity MREs(such as homologous parental gene and pseudogene) have high competition to ‘sponge’ miRNA leading to a substantial influence on its rivals. In contrast, low‐affinity MREs have a little or no influence on peer RNAs. If MREs are equal in affinity for a given miRNA, a larger population of ceRNAs has a weaker influence on each other when competing for binding of the shared miRNA. On the other hand, when the population is small enough (such as comprised of only two MREs) the competitive effect on each other will be detectable. Thus, increase of pseudogene expression may decrease repression of miRNAs on parental genes.

## Perspective

Taking into account all transcripts as a whole network for the analysis of biological function, scientists eventually need to identify all spatiotemporally alterations in MREs within the context of the whole transcriptome. It is not enough to enhance or knock‐down one miRNA/ceRNA expression to observe biological function change, because single miRNA/ceRNA may play a very subtle function in the entire network. A group of relevant transcripts may be integrated together as one object to analyse their function. Many protein‐coding genes have pseudogenes and many have more than one pseudogene remaining through evolution 68. Functional analysis of pseudogenes should be integrated into an entire network of RNAs where the role of each transcript can be properly determined. Indeed, this is an enormous undertaking and is unlikely to be resolved in the near future. Furthermore, considering different sub‐cellular location within specific organelles, some local pseudogene transcripts or ceRNA network may be present in sub‐cellular compartments. This will add another level of complexity in understanding roles of pseudogene transcripts in the entire ceRNA network.

Available data indicated that interaction among the ceRNA network is involved in diverse biological functions. Due to imperfect pairing of miRNA with target MREs, the regulatory nature of pseudogenes *via* ceRNA cannot simply be derived from genomic sequence. Based on sequence homology, pseudogenes may play biological functions similar to lncRNAs which have been well reviewed 69. In addition to competitive binding of shared RNAs, pseudogene transcripts are likely to compete for association with RNA binding proteins. This would then interfere with the potential role of RNA binding proteins in regulating splicing or stability of the parental genes. It remains unclear whether pseudogenes compete for transcriptional modulators or transcription factors which could also affect parental gene expression at the transcriptional level 8. In the future, it will be important to explore the ability of pseudogenes to modulate parental gene expression at multiple levels under different cellular conditions.

## Conflicts of interest

All the authors confirm that there are no conflicts of interest.

## References

[1] Lundstrom K . Micro‐RNA in disease and gene therapy. Curr Drug Discov Technol. 2011; 8: 76–86. 21513487 10.2174/157016311795563857

[2] Bosson AD , Zamudio JR , Sharp PA . Endogenous miRNA and target concentrations determine susceptibility to potential ceRNA competition. Mol Cell. 2014; 56: 347–59. 25449132 10.1016/j.molcel.2014.09.018 PMC5048918

[3] Salmena L , Poliseno L , Tay Y , *et al* A ceRNA hypothesis: the Rosetta Stone of a hidden RNA language? Cell. 2011; 146: 353–8. 21802130 10.1016/j.cell.2011.07.014 PMC3235919

[4] Qu S , Yang X , Li X , *et al* Circular RNA: a new star of noncoding RNAs. Cancer Lett. 2015; 365: 141–8. 26052092 10.1016/j.canlet.2015.06.003

[5] Wang J , Liu X , Wu H , *et al* CREB up‐regulates long non‐coding RNA, HULC expression through interaction with microRNA‐372 in liver cancer. Nucleic Acids Res. 2010; 38: 5366–83. 20423907 10.1093/nar/gkq285 PMC2938198

[6] Karreth FA , Pandolfi PP . ceRNA cross‐talk in cancer: when ce‐bling rivalries go awry. Cancer Discov. 2013; 3: 1113–21. 24072616 10.1158/2159-8290.CD-13-0202 PMC3801300

[7] Xiao‐Jie L , Ai‐Mei G , Li‐Juan J , *et al* Pseudogene in cancer: real functions and promising signature. J Med Genet. 2015; 52: 17–24. 25391452 10.1136/jmedgenet-2014-102785

[8] Grander D , Johnsson P . Pseudogene‐expressed RNAs: emerging roles in gene regulation and disease. Curr Top Microbiol Immunol. 2016; 394: 111–26. 25982975 10.1007/82_2015_442

[9] Kalyana‐Sundaram S , Kumar‐Sinha C , Shankar S , *et al* Expressed pseudogenes in the transcriptional landscape of human cancers. Cell. 2012; 149: 1622–34. 22726445 10.1016/j.cell.2012.04.041 PMC3597446

[10] Porter KA , Duffy EB , Nyland P , *et al* The CLRX.1/NOD24 (NLRP2P) pseudogene codes a functional negative regulator of NF‐kappaB, pyrin‐only protein 4. Genes Immun. 2014; 15: 392–403. 24871464 10.1038/gene.2014.30 PMC4311403

[11] Ji Z , Song R , Regev A , *et al* Many lncRNAs, 5′UTRs, and pseudogenes are translated and some are likely to express functional proteins. Elife. 2015; 4: e08890. 26687005 10.7554/eLife.08890 PMC4739776

[12] Xu J , Zhang J . Are human translated pseudogenes functional? Mol Biol Evol. 2016; 33: 755–60. 26589994 10.1093/molbev/msv268 PMC5009996

[13] Guo X , Deng L , Deng K , *et al* Pseudogene PTENP1 suppresses gastric cancer progression by modulating PTEN. Anticancer Agents Med Chem. 2016; 16: 456–64. 25968876 10.2174/1871520615666150507121407

[14] Poliseno L , Salmena L , Zhang J , *et al* A coding‐independent function of gene and pseudogene mRNAs regulates tumour biology. Nature. 2010; 465: 1033–8. 20577206 10.1038/nature09144 PMC3206313

[15] Yu G , Yao W , Gumireddy K , *et al* Pseudogene PTENP1 functions as a competing endogenous RNA to suppress clear‐cell renal cell carcinoma progression. Mol Cancer Ther. 2014; 13: 3086–97. 25249556 10.1158/1535-7163.MCT-14-0245 PMC4265235

[16] Djebali S , Davis CA , Merkel A , *et al* Landscape of transcription in human cells. Nature. 2012; 489: 101–8. 22955620 10.1038/nature11233 PMC3684276

[17] Furuno M , Pang KC , Ninomiya N , *et al* Clusters of internally primed transcripts reveal novel long noncoding RNAs. PLoS Genet. 2006; 2: e37. 16683026 10.1371/journal.pgen.0020037 PMC1449886

[18] Core LJ , Waterfall JJ , Lis JT . Nascent RNA sequencing reveals widespread pausing and divergent initiation at human promoters. Science. 2008; 322: 1845–8. 19056941 10.1126/science.1162228 PMC2833333

[19] Han L , Yuan Y , Zheng S , *et al* The Pan‐Cancer analysis of pseudogene expression reveals biologically and clinically relevant tumour subtypes. Nat Commun. 2014; 5: 3963. 24999802 10.1038/ncomms4963 PMC4339277

[20] Milligan MJ , Lipovich L . Pseudogene‐derived lncRNAs: emerging regulators of gene expression. Frontiers in genetics. 2014; 5: 476. 25699073 10.3389/fgene.2014.00476 PMC4316772

[21] Salmena L . Pseudogene redux with new biological significance. Methods Mol Biol. 2014; 1167: 3–13. 24823767 10.1007/978-1-4939-0835-6_1

[22] Elliott KT , Cuff LE , Neidle EL . Copy number change: evolving views on gene amplification. Future Microbiol. 2013; 8: 887–99. 23841635 10.2217/fmb.13.53

[23] Hajjari M , Salavaty A . HOTAIR: an oncogenic long non‐coding RNA in different cancers. Cancer Biol Med. 2015; 12: 1–9. 25859406 10.7497/j.issn.2095-3941.2015.0006 PMC4383848

[24] Ling H , Vincent K , Pichler M , *et al* Junk DNA and the long non‐coding RNA twist in cancer genetics. Oncogene. 2015; 34: 5003–11. 25619839 10.1038/onc.2014.456 PMC4552604

[25] Davis AP , Benninghoff AD , Thomas AJ , *et al* DNA methylation of the LIN28 pseudogene family. BMC Genom. 2015; 16: 287. 10.1186/s12864-015-1487-3 PMC4404226 25884154

[26] Chan WL , Chang JG . Pseudogene‐derived endogenous siRNAs and their function. Methods Mol Biol. 2014; 1167: 227–39. 24823781 10.1007/978-1-4939-0835-6_15

[27] Poliseno L , Pandolfi PP . PTEN ceRNA networks in human cancer. Methods. 2015; 77–78: 41–50. 10.1016/j.ymeth.2015.01.013 25644446

[28] Johnsson P , Ackley A , Vidarsdottir L , *et al* A pseudogene long‐noncoding‐RNA network regulates PTEN transcription and translation in human cells. Nat Struct Mol Biol. 2013; 20: 440–6. 23435381 10.1038/nsmb.2516 PMC3618526

[29] Ye X , Fan F , Bhattacharya R , *et al* VEGFR‐1 pseudogene expression and regulatory function in human colorectal cancer cells. Mol Cancer Res. 2015; 13: 1274–82. 26041938 10.1158/1541-7786.MCR-15-0061 PMC4573265

[30] Hawkins PG , Morris KV . Transcriptional regulation of Oct4 by a long non‐coding RNA antisense to Oct4‐pseudogene 5. Transcription. 2010; 1: 165–75. 21151833 10.4161/trns.1.3.13332 PMC2999937

[31] Nakano M , Fukushima Y , Yokota S , *et al* CYP2A7 pseudogene transcript affects CYP2A6 expression in human liver by acting as a decoy for miR‐126. Drug Metab Dispos. 2015; 43: 703–12. 25710939 10.1124/dmd.115.063255

[32] Okamura K , Phillips MD , Tyler DM , *et al* The regulatory activity of microRNA* species has substantial influence on microRNA and 3′ UTR evolution. Nat Struct Mol Biol. 2008; 15: 354–63. 18376413 10.1038/nsmb.1409 PMC2698667

[33] Zheng L , Li X , Gu Y , *et al* The 3′UTR of the pseudogene CYP4Z2P promotes tumor angiogenesis in breast cancer by acting as a ceRNA for CYP4Z1. Breast Cancer Res Treat. 2015; 150: 105–18. 25701119 10.1007/s10549-015-3298-2

[34] Scarola M , Comisso E , Pascolo R , *et al* Epigenetic silencing of Oct4 by a complex containing SUV39H1 and Oct4 pseudogene lncRNA. Nat Commun. 2015; 6: 7631. 26158551 10.1038/ncomms8631 PMC4510692

[35] Marques AC , Tan J , Lee S , *et al* Evidence for conserved post‐transcriptional roles of unitary pseudogenes and for frequent bifunctionality of mRNAs. Genome Biol. 2012; 13: R102. 23153069 10.1186/gb-2012-13-11-r102 PMC3580494

[36] Ala U , Karreth FA , Bosia C , *et al* Integrated transcriptional and competitive endogenous RNA networks are cross‐regulated in permissive molecular environments. Proc Natl Acad Sci USA. 2013; 110: 7154–9. 23536298 10.1073/pnas.1222509110 PMC3645534

[37] Karreth FA , Ala U , Provero P , *et al* Pseudogenes as competitive endogenous RNAs: target prediction and validation. Methods Mol Biol. 2014; 1167: 199–212. 24823779 10.1007/978-1-4939-0835-6_13

[38] Figliuzzi M , Marinari E , De Martino A . MicroRNAs as a selective channel of communication between competing RNAs: a steady‐state theory. Biophys J. 2013; 104: 1203–13. 23473503 10.1016/j.bpj.2013.01.012 PMC3870798

[39] Chiu HS , Llobet‐Navas D , Yang X , *et al* Cupid: simultaneous reconstruction of microRNA‐target and ceRNA networks. Genome Res. 2015; 25: 257–67. 25378249 10.1101/gr.178194.114 PMC4315299

[40] Brugaletta S , Gomez‐Lara J , Serruys PW , *et al* Serial *in vivo* intravascular ultrasound‐based echogenicity changes of everolimus‐eluting bioresorbable vascular scaffold during the first 12 months after implantation insights from the ABSORB B trial. JACC Cardiovasc Interv. 2011; 4: 1281–9. 22192369 10.1016/j.jcin.2011.08.014

[41] Li JH , Liu S , Zhou H , *et al* starBase v2.0: decoding miRNA‐ceRNA, miRNA‐ncRNA and protein‐RNA interaction networks from large‐scale CLIP‐Seq data. Nucleic Acids Res. 2014; 42: D92–7. 24297251 10.1093/nar/gkt1248 PMC3964941

[42] Shao T , Wu A , Chen J , *et al* Identification of module biomarkers from the dysregulated ceRNA‐ceRNA interaction network in lung adenocarcinoma. Mol BioSyst. 2015; 11: 3048–58. 26325208 10.1039/c5mb00364d

[43] Hansen TB , Jensen TI , Clausen BH , *et al* Natural RNA circles function as efficient microRNA sponges. Nature. 2013; 495: 384–8. 23446346 10.1038/nature11993

[44] Memczak S , Jens M , Elefsinioti A , *et al* Circular RNAs are a large class of animal RNAs with regulatory potency. Nature. 2013; 495: 333–8. 23446348 10.1038/nature11928

[45] Ebert MS , Neilson JR , Sharp PA . MicroRNA sponges: competitive inhibitors of small RNAs in mammalian cells. Nat Methods. 2007; 4: 721–6. 17694064 10.1038/nmeth1079 PMC3857099

[46] Yip DK , Pang IK , Yip KY . Systematic exploration of autonomous modules in noisy microRNA‐target networks for testing the generality of the ceRNA hypothesis. BMC Genom. 2014; 15: 1178. 10.1186/1471-2164-15-1178 PMC4367885 25539629

[47] Denzler R , Agarwal V , Stefano J , *et al* Assessing the ceRNA hypothesis with quantitative measurements of miRNA and target abundance. Mol Cell. 2014; 54: 766–76. 24793693 10.1016/j.molcel.2014.03.045 PMC4267251

[48] Broderick JA , Zamore PD . Competitive endogenous RNAs cannot alter microRNA function *in vivo* . Mol Cell. 2014; 54: 711–3. 24905003 10.1016/j.molcel.2014.05.023

[49] Mullokandov G , Baccarini A , Ruzo A , *et al* High‐throughput assessment of microRNA activity and function using microRNA sensor and decoy libraries. Nat Methods. 2012; 9: 840–6. 22751203 10.1038/nmeth.2078 PMC3518396

[50] Tay Y , Rinn J , Pandolfi PP . The multilayered complexity of ceRNA crosstalk and competition. Nature. 2014; 505: 344–52. 24429633 10.1038/nature12986 PMC4113481

[51] Khan I , Kerwin J , Owen K , *et al* Correction: registered report: a coding‐independent function of gene and pseudogene mRNAs regulates tumour biology. Elife. 2015; 4: e13015. 26599840 10.7554/eLife.13015 PMC4656975

[52] Poliseno L , Marranci A , Pandolfi PP . Pseudogenes in human cancer. Front Med (Lausanne). 2015; 2: 68. 26442270 10.3389/fmed.2015.00068 PMC4585173

[53] Guo G , Kang Q , Zhu X , *et al* A long noncoding RNA critically regulates Bcr‐Abl‐mediated cellular transformation by acting as a competitive endogenous RNA. Oncogene. 2015; 34: 1768–79. 24837367 10.1038/onc.2014.131

[54] Rutnam ZJ , Du WW , Yang W , *et al* The pseudogene TUSC2P promotes TUSC2 function by binding multiple microRNAs. Nat Commun. 2014; 5: 2914. 24394498 10.1038/ncomms3914 PMC3896787

[55] Yang J , Li T , Gao C , *et al* FOXO1 3′UTR functions as a ceRNA in repressing the metastases of breast cancer cells via regulating miRNA activity. FEBS Lett. 2014; 588: 3218–24. 25017439 10.1016/j.febslet.2014.07.003

[56] Yang W , Du WW , Li X , *et al* Foxo3 activity promoted by non‐coding effects of circular RNA and Foxo3 pseudogene in the inhibition of tumor growth and angiogenesis. Oncogene. 2016; 35: 3919–31. 26657152 10.1038/onc.2015.460

[57] Peng H , Ishida M , Li L , *et al* Pseudogene INTS6P1 regulates its co gnate gene INTS6 through competitive binding of miR‐17‐5p in hepatocellular carcinoma. Oncotarget. 2015; 6: 5666–77. 25686840 10.18632/oncotarget.3290 PMC4467393

[58] Welch JD , Baran‐Gale J , Perou CM , *et al* Pseudogenes transcribed in breast invasive carcinoma show subtype‐specific expression and ceRNA potential. BMC Genom. 2015; 16: 113. 10.1186/s12864-015-1227-8 PMC4344757 25765044

[59] Jingsi T , Mingyao Y , Ying L . Functional roles of pseudogenes in cancers. Yi chuan. 2015; 37: 8–16. 25608808 10.16288/j.yczz.2015.01.002

[60] Karreth FA , Reschke M , Ruocco A , *et al* The BRAF pseudogene functions as a competitive endogenous RNA and induces lymphoma *in vivo* . Cell. 2015; 161: 319–32. 25843629 10.1016/j.cell.2015.02.043 PMC6922011

[61] Fang L , Du WW , Yang X , *et al* Versican 3′‐untranslated region (3′‐UTR) functions as a ceRNA in inducing the development of hepatocellular carcinoma by regulating miRNA activity. FASEB J. 2013; 27: 907–19. 23180826 10.1096/fj.12-220905

[62] Esposito F , De Martino M , D'Angelo D , *et al* HMGA1‐pseudogene expression is induced in human pituitary tumors. Cell Cycle. 2015; 14: 1471–5. 25894544 10.1080/15384101.2015.1021520 PMC4615119

[63] Esposito F , De Martino M , Forzati F , *et al* HMGA1‐pseudogene overexpression contributes to cancer progression. Cell Cycle. 2014; 13: 3636–9. 25483074 10.4161/15384101.2014.974440 PMC4613653

[64] Wang L , Guo ZY , Zhang R , *et al* Pseudogene OCT4‐pg4 functions as a natural micro RNA sponge to regulate OCT4 expression by competing for miR‐145 in hepatocellular carcinoma. Carcinogenesis. 2013; 34: 1773–81. 23615404 10.1093/carcin/bgt139

[65] Hayashi H , Arao T , Togashi Y , *et al* The OCT4 pseudogene POU5F1B is amplified and promotes an aggressive phenotype in gastric cancer. Oncogene. 2015; 34: 199–208. 24362523 10.1038/onc.2013.547

[66] Ergun S , Oztuzcu S . Oncocers: ceRNA‐mediated cross‐talk by sponging miRNAs in oncogenic pathways. Tumour Biol. 2015; 36: 3129–36. 25809705 10.1007/s13277-015-3346-x

[67] Xu J , Li Y , Lu J , *et al* The mRNA related ceRNA‐ceRNA landscape and significance across 20 major cancer types. Nucleic Acids Res. 2015; 43: 8169–82. 26304537 10.1093/nar/gkv853 PMC4787795

[68] Frankish A , Harrow J . GENCODE pseudogenes. Methods Mol Biol. 2014; 1167: 129–55. 24823776 10.1007/978-1-4939-0835-6_10

[69] Shi X , Nie F , Wang Z , *et al* Pseudogene‐expressed RNAs: a new frontier in cancers. Tumour Biol. 2016; 37: 1471–8. 26662308 10.1007/s13277-015-4482-z

